# Application of Statistical Methods to Accurately Assess the Effect of Gamma Aluminum Oxide Nanopowder on the Hardness of Composite Materials with Polyester–Glass Recyclate

**DOI:** 10.3390/ma15175957

**Published:** 2022-08-29

**Authors:** Norbert Abramczyk, Sebastian Drewing, Katarzyna Panasiuk, Daria Żuk

**Affiliations:** Faculty of Marine Engineering, Gdynia Maritime University, 81225 Gdynia, Poland

**Keywords:** nanoadditives, nanocomposites, recycling, hardness, statistical analysis

## Abstract

Polymer composites are materials that are used in many industries. Their wide application has a direct impact on the amount of post-production and post-consumer waste. The global problem with recycling, especially of fiber-reinforced polymeric materials, has prompted research into methods of their use. Previous research on composite materials with polyester–glass recyclate showed a decrease in mechanical properties. The construction material should have the highest mechanical properties. Based on the literature, it was found that the use of nanoadditives may have a positive effect on the parameters of the materials. The use of gamma aluminum nanopowder, in a small amount can significantly increase the mechanical properties of composites with polyester–glass recyclate, and thus can affect the application of these materials to structural elements. The article is devoted to the research on the hardness of composite materials with polyester–glass recyclate and gamma aluminum nanopowder. The main goal is to investigate the possibility of using a nanoadditive as a material, increasing the mechanical properties of composites with polyester–glass recyclate, so as to create a recycled material with the highest possible strength parameters. Hardness tests were performed using the Barcol method. For each composite material, 30 measurements were made in order to subject the results to a statistical analysis. Using parametric statistical tests it was shown that the obtained hardness values at the assumed level of statistical significance *p_v_* = 0.05 for comparisons for the samples of the reference material (B0) do not differ by chance, while for the comparisons in the configurations of the reference material (B0) with the modified materials, (R10, A2, R10A2) they do not differ by accident. Studies have shown that the addition of 2% gamma aluminum nanopowder slightly lowers the hardness of a pure polyester–glass composite, but the same additive allows the hardness of composite materials to be increased with the addition of glass recyclate. This is of particular importance for the development of the optimal composition of polyester–glass composites with the addition of recyclate, which will have good strength properties and at the same time enable the reuse of composite waste.

## 1. Introduction

Composite materials, and in particular polyester–glass laminates reinforced with fibers, are materials that are becoming more and more popular, and thus the production of elements based on them has increased. They are the main construction material in the yachting and shipbuilding industries, as well as in railways, automation and aviation [1,2,3]. These materials are the subject of research by scientists around the world, from the mechanical research of new materials [4] to tribological research [5,6]. The use of these materials on an increasing scale does not eliminate the problem of the utilization and recycling of this material. Previous studies [7], aimed at designing, manufacturing and determining the mechanical properties of a material with a filler in the form of polyester–glass recyclate, which could provide a closed circuit in the yacht industry, showed a significant decrease in mechanical properties. In order to increase the properties, research was directed towards nanofillers. The properties of composite materials depend not only on the physicochemical properties of the components, but also on the size of the contact surface of the dispersed phase (filler) and the nature of interactions between the continuous and dispersed phases [8]. The mechanical properties of composites increase with an increase in the aspect ratio of the filler and a decrease in its transverse dimension [9,10]. Nanocomposites have a number of advantages, including high values of the modulus of elasticity, high impact strength, high deformability, good short-term and fatigue strength characteristics, increased fire resistance, increased resistance and thermal stability [8,11,12,13].

Powder nanoadditives have a beneficial effect on the mechanical properties of nanocomposites; however, they are used in many industries [14,15,16]. The article [17] uses the ultra-high sensitive V3.6Mo2.4O16-chitosan (MV-CHT) nanocomposite to obtain an electrochemical sensor for drug monitoring. The research [18] used magnetic CoFe_2_O_4_ nanoparticles to produce hydrogel beads as drug encapsulation and release systems. In the research [19], Al_2_O_3_ nanopowder was used to create polyurethane nanocomposites. These materials were characterized by increased stiffness, hardness and mechanical strength. In the case of polymethyl methacrylate (PMMA), after adding 5 wt. of Al_2_O_3_ nanoparticles, an increase of 28% in the elongation at break was found in relation to pure PMMA. When analyzing the hardness results, it was found that it increases by 8%, regardless of the percentage of nanofillers used [19]. In the paper [20], which analyzed the influence of γ-Al_2_O_3_ nanoparticles on the epoxy resin itself, an improvement in its stiffness and strength was found. In studies of metal matrix composites reinforced with nanoparticles, an increase in hardness by 5–16 HRB, bending strength by 54%, wear resistance and a decrease in the friction coefficient were found [21]. In the studies of scientific research, statistical tests are used to check the normality of the distribution of the examined feature and are applicable in the study of multivariate samples [22].

When developing research results, it is also extremely important to choose the appropriate method of statistical analysis [23]. It is important to choose an appropriate method of processing statistical data and to use knowledge competently in various research activities [24,25,26].

The hardness tests of composite materials with polyester–glass recyclate showed, in accordance with the rest of the mechanical tests, an increase in hardness along with an increase in the percentage of filler in the form of the recyclate and 2% of gamma -aluminum nanopowder and are directly related to previous studies [1,3,4,7,27]. Statistical analysis of the results of hardness measurements made it possible to authenticate the non-randomness of the results.

## 2. Materials and Methods

The materials used for the research were polyester–glass laminates with the addition of a non-filler in the form of gamma aluminum nanopowder. Composite materials were made by hand lamination. Polyester resin Polimal 1094-AWTP was used, as well as glass mat with a weight of 450 g/m^2^. The recyclate used in the research is polyester–glass waste ground on a granulation sieve ≤ 1.2 mm. The matrix additives, such as recyclate filler and nanofiller, were combined by physical mixing using a stirrer. The produced materials are: B0 glass mat (10 layers), polyester resin; base R10 glass mat (10 layers), polyester resin, 10% polyester–glass recyclate; with A2 nanopowder glass mat (10 layers), polyester resin, 2% gamma aluminum nanopowder; R10A2 glass mat (10 layers), polyester resin, 10% polyester–glass recyclate, 2% gamma aluminum nanopowder. Due to the addition of an aluminum nanoadditive to the composite already with the addition of recyclate, 2% was chosen to verify the effect at small amounts. It also has an economic aspect, as nanoadditives are not easily obtained, which has an impact on the price. Adding in greater amounts to the composite would affect the price of the end product as well as the cost-effectiveness of production. The weight percentages are shown in Table 1.

**Figure 1:**
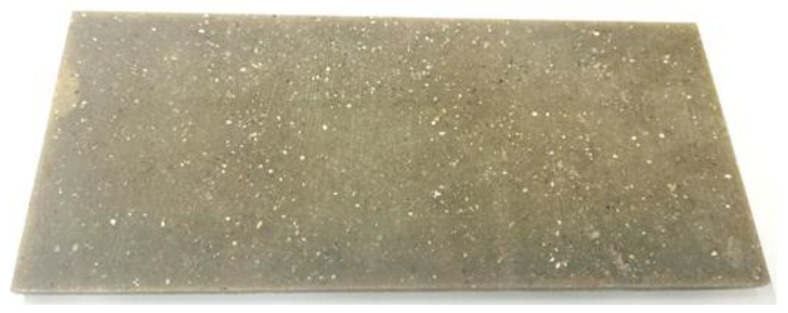
The sample prepared for hardness tests, R10, dimensions 300 × 100.

The tests were carried out on 4 samples in order to thoroughly verify the influence of the nanoadditive on the mechanical properties of the composite. The test samples were prepared using the water cutting method. Figure 1 shows a sample prepared for hardness tests.

Measurements of the hardness of plastics are made for design and research purposes, as well as to control the quality of products. Hardness tests were carried out using the Barcol method (PN-EN 59: 2016-03). In this method, the measurement is carried out using a measuring pin under the load of a spring, pressed into a sample placed on the substrate, obtaining a reading on a scale from 1–100. Apart from presenting the obtained results, statistical methods were used to better visualize the processes taking place in the material. In order to calculate the normal distribution of the data, the Shapiro–Wilk test was used, recommended for use with a sample size less than 30.

## 3. Results

### 3.1. Hardness Measurements of Tested Samples

**Figure 2:**
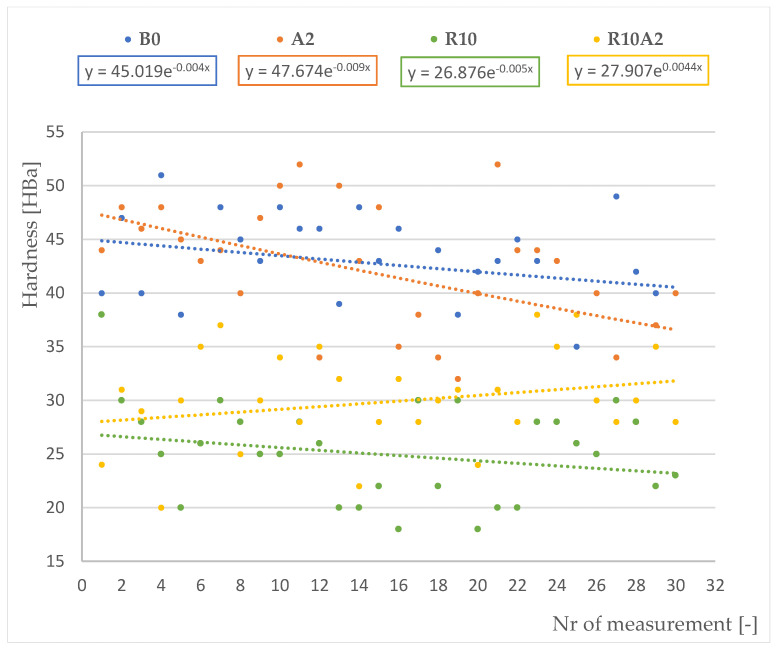
Hardness test results for four variants of composite materials.

Table 2 shows the average hardness values of the tested composite materials with the use of the Barcol hardness tester. Figure 2 shows a graphic illustration of the hardness measurements for each composite variant.

For each composite material, 30 measurements were made using both methods in order to statistically analyze the results. Studies have shown that the addition of 2% gamma aluminum nanopowder slightly reduces the hardness of composites without the addition of recyclate. The hardness of the base material was 43 HBa, and with the addition of 2% gamma aluminum nanopowder to the base material, the hardness decreased by 2.3%. The addition of 10% polyester glass recyclate to the base composite adversely affects the hardness of the obtained material. The hardness of the R10 composite decreased by 42% compared to the base composite B0. It is worth noting that the modifications in the form of the addition of 2% gamma aluminum nanopowder to the R10 composite allows the value of the obtained R10A2 composite to be increased—in this case the decrease in hardness compared to the base material is 29%.

### 3.2. Statistical Analysis

According to the operating manual of the Barcol 934-1 hardness tester for glass fiber-reinforced plastics, the number of measurements according to GB/T 3854-2005 for a hardness of 30 on the HBA scale is 29. Due to the anisotropy and a large range of values of the obtained results, it was decided to determine the sample size on the basis of a computational model for the mean with known population standard deviation [28].

(1) $$n\geq {\left(u_{1-\frac{\alpha }{2}}\cdot \frac{\sigma }{d}\right)}^{2}$$

where: *σ* is the standard deviation, *d* is the maximum allowable measurement error, *α* is the significance level, $u_{1-\frac{\alpha }{2}}$ is the critical value read from the normal distribution table.

To determine the sample size, data from the test of a 10% recyclate sample with the addition of aluminum (10% from aluminum)–sample R10A2 were adopted.

Thus, the parametric Student’s *t*-tests and ANOVA were used for statistical analyses. We calculated: *σ* = 2.83, *d* = 0.5 and $u_{1-\frac{\alpha }{2}}$ = 0.97 for the significance level *α* = 95%.

According to the Formula (1), the sample size n was equal to 30.94. *n* = 30 was assumed for the tests, and the calculated value of the sample size is the same as that indicated in the instruction manual of the device.

In order to determine the type of statistical test, the normality of distributions was tested [29]. The obtained values of the probabilities *p* of the Shapiro–Wilk normality tests (tests with the highest power for samples with a small number of up to *n* = 2000) [30,31] for the individual four samples of the tested material (Table 3) showed that the distributions are normal (the calculated *p*-values are greater than the assumed *p_v_* = 0.05).

**Figure 3:**
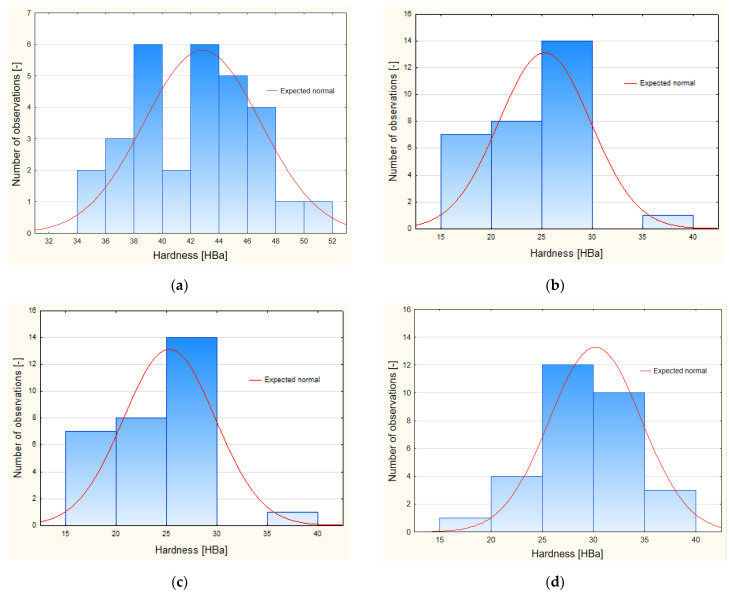
Histogram hardness HBa for: (**a**) sample B0, Shapiro–Wilk W = 0.97431, *p* = 0.66237, x ≤ class boundary; (**b**) sample A2, Shapiro–Wilk W = 0.96963, *p* = 0.52897, x ≤ class boundary; (**c**) sample R10, Shapiro–Wilk W = 0.93655, *p* = 0.07345, x ≤ class boundary; (**d**) sample R10A2, Shapiro–Wilk W = 0.96540, *p* = 0.42213, x ≤ class boundary.

Figure 3 shows the values of the probabilities *p* of the Shapiro–Wilk normality tests for individual samples of the tested material.

On the basis of the analysis, it is noticeable that in the case of some samples, the results differ significantly from the normal expected. The distribution of the probabilities *p* of the Shapiro–Wilk test is the same in the case of samples B0 and R10A2, and in the case of other samples, these results partially overlap.

#### 3.2.1. Dependent Variable Test

For these comparisons, it was assumed that the B0 sample is the state before the interference (standard), and the remaining samples with recyclate and aluminum additives are the state after the interference. A dependent sample is the study of the same group under different conditions, after an interference. In practice, the mean values in the two groups will always be different. An important role of the test, however, is to indicate whether these differences are statistically significant or whether they are accidental, i.e., “differ by chance”. If the distribution of a population trait is the normal distribution, the Student’s t-test is the most powerful. The values of the obtained results are presented in Table 4.

**Figure 4:**
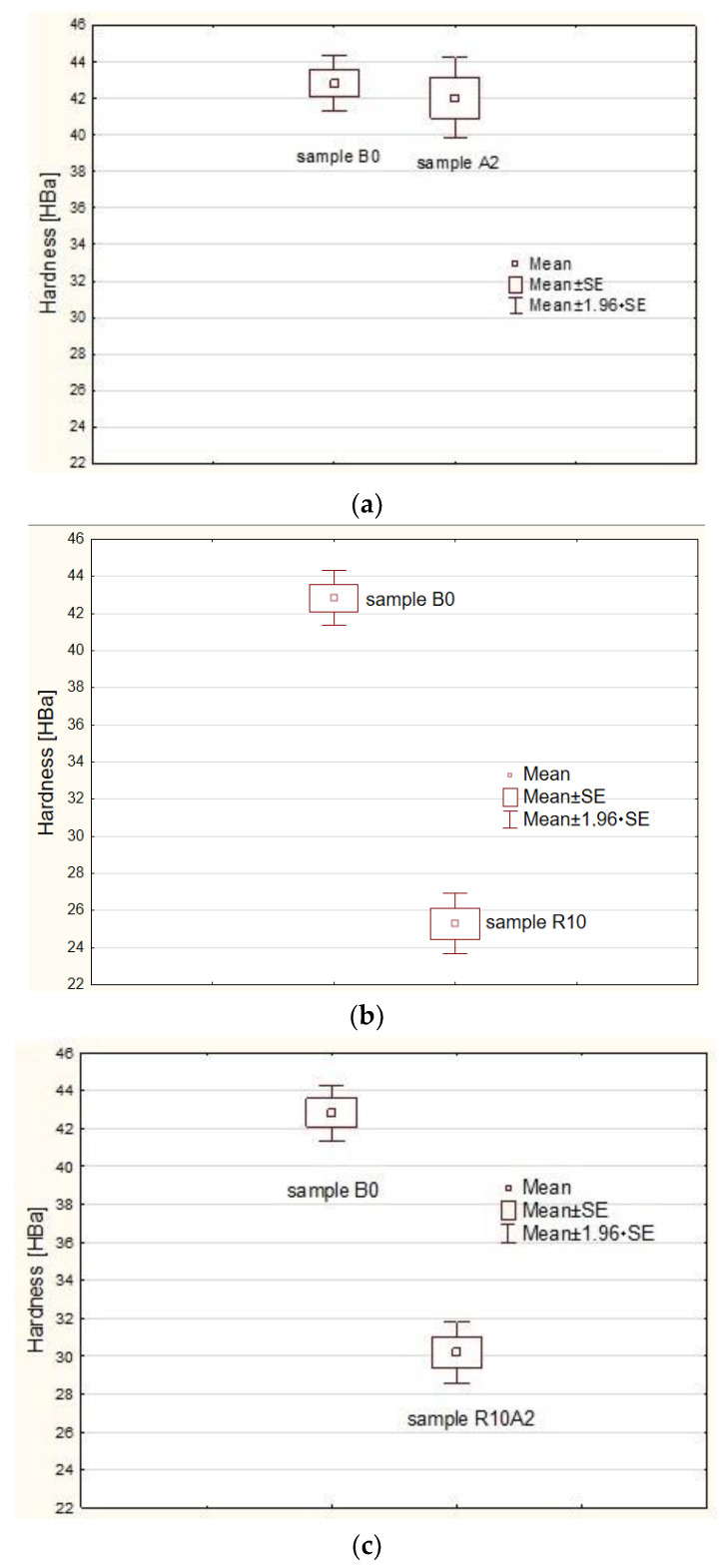
Frame-whisker chart for: (**a**) sample B0 versus sample A2; (**b**) sample B0 versus sample R10; (**c**) sample B0 versus sample R10A2, where: mean ± SE was mean ± standard error and mean ± 1.96 × SE was confidence interval for the mean.

Figure 4 shows the frame-whisker chart for the three considered comparisons of the examined composite samples.

**Figure 5:**
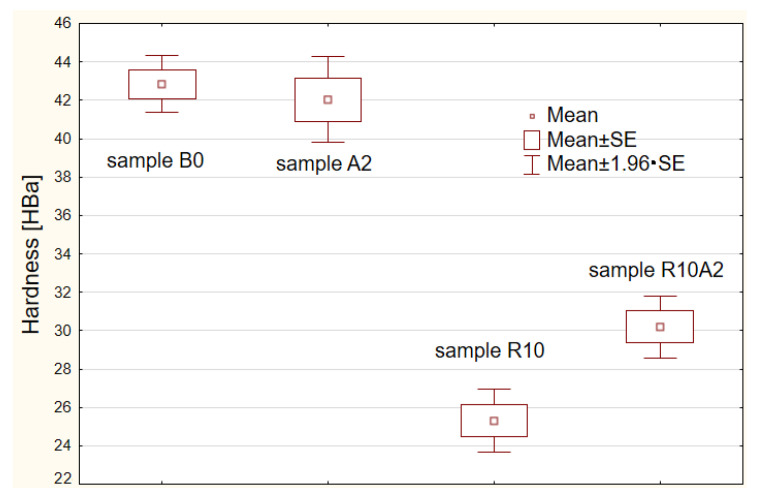
Multiple comparison chart for individual samples where: mean ± SE was mean ± standard error and mean ± 1.96 × SE was confidence interval for the mean.

The obtained *p*-values of the pairwise comparisons (Table 4) and the box-whisker plots (Figure 4 and Figure 5) showed that only the B0 and A2 samples did not differ significantly (the calculated *p* = 0.525898 is greater than the assumed *p_v_* = 0.05). There are significant statistical differences between the remaining samples.

#### 3.2.2. Independent Variable Test

For these comparisons it was assumed that all samples were independent and made of different materials. Multiple comparison ANOVA tests were used for the comparisons. A prerequisite for the production of ANOVA statistics is the homogeneity of variance test. The Brown–Forsythe test was used to verify the hypothesis of homogeneity of the variance of the studied variable in several (*n* ≥ 2) populations. The calculated value of the Brown–Forsythe test, *p* = 0.1117, allows for the null hypothesis of homogeneity of variance. Therefore, it was decided to perform ANOVA parametric statistics for all tested samples. As a post hoc test, the Scheffé test was chosen as it is considered the most conservative, where it is more difficult to achieve significant differences between samples than with other tests. The results obtained from the above analyses are presented in Table 5. 

Figure 5 shows a graph of the comparisons made with the use of ANOVA statistics.

The obtained *p*-values of multiple ANOVA comparisons (Table 5) and the box-whisker plots showed that only the B0 and A2 samples did not differ significantly (the calculated *p* = 0.94045 is greater than the assumed *p_v_* = 0.05). There are significant statistical differences between the remaining samples.

#### 3.2.3. Determination of the Anisotropy of Samples on the Basis of the Classical Coefficient of Variation

In order to find differences in the anisotropy of the surface layer of the tested samples, their statistical analysis was conducted on the basis of the classical coefficient of variation being the quotient of the standard deviation of the feature and its arithmetic mean. This coefficient belongs to the category of relative measures of variation, i.e., measures that depend on the average value of the examined feature. The use of the coefficient of variation made the difference in the dispersion of the hardness values for the visible tested materials. The values of the classical coefficient of variation Vs were calculated from the formula [32]

(2) $$V_{s}=\frac{\sigma }{\bar{x}}\cdot 100\%$$

where: *σ* is the standard deviation and $\bar{x}$ is the arithmetic mean of the variable value.

The coefficient of variation is a unitless measure (expressed as a percentage) and this property makes it possible to compare the diversity of various statistical features. The coefficient of variation tells you how the results are dispersed with respect to how large the average is. This allowed for: (a) determination of the relative measures of dispersion and (b) comparison of the variability of hardness for many samples. The following interpretation of the *V_s_* coefficient was adopted: (a) <26%—low variability, (b) (26–45%)—average variability, (c) (46–100%)—strong variability, (d) >100%—very strong volatility. Table 6 presents the values of the coefficient of variation Vs for the tested materials.

The values of the classical coefficient of variation *V_s_*, calculated according to Formula (2) (Table 4), showed a *V_s_* < 26% for all tested materials, i.e., there was a small variation in the hardness of the tested surface layer. The smallest variation was for the reference sample, i.e., B0, and the greatest for the R10 sample.

## 4. Discussion

Composite materials are often used in the electrical industries. These materials are characterized by a high versatility and a wide range of application possibilities. The research [33] describes the possibilities of using the properties of composites and their modeling in order to obtain the required parameters for a specific application. Such modifications can be made in two main ways: physically or chemically. Modification of a given composite achieved by chemical action may be associated with the addition of an additional component associated with a chemical reaction, copolymerization or functionalization. 

The article [34] presents an analysis of the impact of introducing aluminum nanoadditive as a reinforcing reagent into the HPMC matrix. The introduction of this additive significantly improved the mechanical and barrier properties of the film. The additives occupy the spaces in the pores of the matrix, which increases the tendency of the pores to collapse and improves the chemical bond between the base material and the additives. The conducted wear tests show that the addition of nanoadditives can improve the tribological performance of the HPMC composite while reducing wear and friction. 

The paper [35] presents an analysis of the use of nanocomposites containing fillers, such as: layered silicates, cellulose nanostrengtheners or carbon nanotubes, as well as silica nanoparticles or starch nanocrystals. The study showed that the use of appropriate nanoadditives enables the production of packaging materials with better strength, mechanical and barrier properties, and also improves other physical properties. Research materials based on polyester resin, glass mat and polyester–glass recyclate obtained in the recycling of composite waste materials were produced for the tests. In order to manage composite waste and pro-ecological activities, materials were obtained with the addition of recyclate, which reduces the strength parameters of the obtained material in relation to the strength properties of materials produced without the use of recyclate. In order to reduce the negative effects of using recyclate, an attempt was made to use the aluminum nanoadditive as a factor enhancing the parameters of the new material with recyclate and to reduce the scope of negative changes resulting from the addition of recyclate. Based on the statistical analysis of the obtained results, it can be concluded that the empirical hardness distributions for all samples are symmetric or normal Gays distributions, which allows justifying the possibility of using parametric statistical tests for comparisons of the results of experimental tests. 

The obtained *p*-values of pairwise comparisons and multiple ANOVA comparisons showed that only samples B0 and A2 did not differ statistically significantly (calculated *p* = 0.5258 and *p* = 0.9404 is greater than the assumed *p_v_* = 0.05). There are significant statistical differences between the remaining samples. This allows the conclusion that the addition of aluminum to the standard sample B0 does not significantly change its hardness. The samples B0, 43 HBa, and A2, 42 HBa, are characterized by the highest hardness. The addition of 2% gamma aluminum nanopowder to the R10 sample significantly increased its hardness. The R10 sample is the most anisotropic, as evidenced by the highest value of the coefficient of variation *V_s_* = 18.013. Sample B0 is the least anisotropic, as evidenced by the lowest value of the coefficient of variation *V_s_* = 9.618. The A2 and R10A2 samples have the same anisotropy, as evidenced by the almost same value of the coefficient of variation *V_s_* = 14.751 and *V_s_* = 14.920.

The analysis of the research results showed a positive effect of the aluminum nanoadditive on the strength parameters of this material. As a result of the use of this additive, an environmentally friendly composite material with higher performance properties was obtained.

## 5. Conclusions

The results of the measurements of the hardness of materials confirmed the assumption that the addition of 2% nanopowder of gamma aluminum has a positive effect on the hardness of polyester–glass composites with the addition of polyester–glass recyclate. Modification of the composition of composites allows a significant impact on the values of mechanical and strength parameters of the material. Glass–polyester composites with the addition of recyclate and the addition of 2% gamma aluminum nanopowder can therefore be an environmentally friendly alternative to ordinary composite materials.

## Figures and Tables

**Table 1:** Percentage (by weight) of individual components.

Sample	Resin %	Matrix %	Recyclate %	Nanoadditive %
B0	60	40	0	0
R10	60	30	10	0
A2	60	38	0	2
R10A2	60	28	10	2

**Table 2:** Hardness of composite materials (average measurement).

Sample	B0	A2	R10	R10A2
Hardness, HBa	43	42	25	30

**Table 3:** The *p*-values of the Shapiro–Wilk distribution normality tests.

Type of Sample	*p*
B0	0.65
A2	0.53
R10	0.07
R10A2	0.42

**Table 4:** *p*-values of Student’s *t*-tests for paired samples.

Lp	Type of Sample	*p*
1	B0 and A2	0.525898
2	B0 and R10	0
3	B0 and R10A2	0

**Table 5:** *p*-values of Scheffé post hoc tests for independent samples.

Type of Sample	Probabilities *p* for Post Hoc (2-Sided) Tests			
	B0	B0	B0	R10
B0		0.9404	0.00000	0.00000
A2	0.9404		0.00000	0.00000
R10	0.00000	0.00000		0.00277
R10A2	0.00000	0.00000	0.00277	

**Table 6:** Values of the coefficient of variation *V_s_* for the tested materials.

Type of Material	*V_s_*
B0	9.6177
A2	14.7515
R10	18.0130
R10A2	14.9203

## Data Availability

The data presented in this study is available upon request to the corresponding author. The data is not publicly available due to too much data.
